# Ethiopian Native Highlander's Adaptation to Chronic High-Altitude Hypoxia

**DOI:** 10.1155/2022/5749382

**Published:** 2022-04-15

**Authors:** Ayechew Getu

**Affiliations:** Department of Physiology, College of Medicine and Health Sciences, University of Gondar, P.O. Box 196, Gondar, Ethiopia

## Abstract

People living in a high-altitude environment have distinct lifelong challenges. Adaptive mechanisms have allowed high-altitude residents to survive in a low-oxygen environment for thousands of years. The purpose of this review was to provide a brief review of the Ethiopian native highlanders' adaptive mechanisms to chronic hypoxia problems at high altitude. Traditionally, an elevated hemoglobin concentration has been considered as a hallmark of lifelong adaptation to high-altitude hypoxia, though this notion has been refuted recently as a result of the establishment of the alternative adaptive responses found in Amhara highlanders living in the Simien Mountains of northern Ethiopia. These populations did not have elevated hemoglobin (no erythrocytosis) but had normal hemoglobin saturation and arterial oxygen level, which alerts researchers to explore the possibility of the presence of an alternative adaptive mechanism. Contrary to this, Oromos living in the Bale Mountains of southern Ethiopia have elevated hemoglobin. The presence of increased nitric oxide (NO) and cyclic guanosine monophosphate (cGMP) in native Amhara highlanders suggests the possibility of adaptation via vasodilation, which would improve oxygen supply to metabolic tissues. Native Amhara highlanders showed no indications of chronic mountain sickness and had a higher pulmonary blood pressure without having a higher pulmonary vascular resistance. In addition, the cerebral circulation is sensitive to NO and carbon dioxide (CO_2_) but not to hypoxia, which would likely promote increased cerebral blood flow and increase oxygen delivery to the brain, making Ethiopian high-altitude natives better suited for survival at high altitudes. Further research is warranted to translate these background natural features of Ethiopian native highlanders to clinical applications.

## 1. Background

High altitude is uniquely characterized by temperature variation, ultraviolet radiation, humidity level, nutrient composition, and most importantly a decrease in atmospheric pressure. Because of this feature of the environmental condition, it is stressful and challenging not only for temporary residents but also for natives. However, human beings have strived in this harsh environment for thousands of years by using genetic and phenotypic adaptive mechanisms.

Exposure to high-altitude challenges and the effects that follow on the body have been known for more than a century [1]. Barometric pressure, along with partial pressure of oxygen, decreases as altitude increases, and ascent to a high altitude above 2500 m is usually associated with the symptoms and signs of a decrement in oxygen supply to metabolic tissues. The degree of symptoms experienced usually increases as the altitude level increases, and often, the symptoms subside upon returning to low altitude [2].

Moreover, besides the observation of symptoms, attaining altitudes high above 3000 m has been associated with anatomical and physiochemical alterations. Early research revealed that because of the presence of long-term adaptive mechanisms, lowlanders can strive and live permanently at an altitude of up to 4900 m, whereas native highlanders can reside at a maximum altitude of 5800–6000 m [3].

It is estimated that about 82 million people live permanently at an altitude of 2500 m or above [4]. The Amhara highlanders of Ethiopia, the Andeans of South America, and the Tibetans of Central Asia are well-known populations that reside in the high altitudes and have thrived for thousands of years. Amharas in the Simien Mountains have been permanently inhabited for more than 70,000 years, whereas in southern Ethiopia, Oromos inhabited the Bale Mountain areas for about 500 years [5, 6].

These people have been well adapted to the hypoxic environmental challenges for thousands of years, and researchers have found different patterns of phenotypic and genotypic adaptive mechanisms that help inhabitants live from generation to generation [5, 7].

## 2. Chronic Effects of High-Altitude Environment on the Human Body

Mountaineers, tourists, and trekkers who normally reside at low altitudes usually experience the acute effects of the ascent to high altitudes. Acute exposure to low oxygen initially changes the mechanics of breathing. There is an increase in the rate and depth of breathing (hyperpnoea), which is eventually associated with a decrease in the level of CO_2_ in the blood (hypocapnia). This low blood CO_2_ level causes respiratory alkalosis, which may be indicative of acute mountain sickness (AMS) [8, 9]. The symptoms of AMS range from mild to severe forms and include sleeping problems, headache, dizziness, nausea and other gastrointestinal disorders, pulmonary edema, and cerebral edema [10].

Acute alterations in heart activity have been linked to rapid exposure to high-altitude hypoxia. The initial response to hypoxia is an increase in cardiac output due to the increase in heart rate and a temporary increase in arterial blood pressure as a result of sympathetic activations [11].

Permanently residing at a high altitude has a unique clinical consequence, which is known as chronic mountain sickness (CMS). It is an indication of the failure of adaptation to chronic hypoxia and is characterized by excessive erythrocytosis, severe hypoxemia, and, in some cases, moderate to severe pulmonary hypertension which may evolve into cor pulmonale, leading to right ventricular failure [12]. The global consensus on the diagnosis of CMS is that there should be an elevation of hemoglobin concentration ≥ 21 g/dl for males or ≥19 g/dl for females together with a minimum score of symptoms [12].

It has been noted that the clinical presentations of CMS increase with increased altitude and the signs and symptoms gradually disappear after descending to low altitude [13].

## 3. Ethiopian Patterns of Adaptations to Chronic High-Altitude Hypoxia

Ethiopia is part of Eastern Africa with varied geographical landscapes ranging from very low altitudes (Afar Triangle, 1000 m below sea level) to high altitudes in the Simien Mountains (the highest peak in Ethiopia and fourth in Africa) in the Amhara region of North Gondar at 4550 m above sea level. Ethiopia is divided into three well-defined geographical regions: the western highlands, the eastern highlands, and the rift valley with the lowland area.

The Simien Mountain regions are the northern part of the western highlands, where the Amharas have lived for millennia at an altitude of 2400–3700 m [5]. Native Amhara highlanders live harmoniously with low oxygen levels. In the present day, researchers found three patterns of adaptations to chronic hypoxia: Ethiopian, Tibetan, and Andean patterns of adaptations [14].

This review is aimed at presenting a brief overview of the Ethiopian native highlander's adaptive mechanisms to chronic high-altitude hypoxic challenges. Relevant literature searches were conducted by using search engines including PubMed and Google Scholar, typing the following keywords: “Ethiopian native highlanders”, “adaptation”, “chronic hypoxia”, and “Siemen Mountains”.

### 3.1. Hematological Adaptations

Traditionally, a high concentration of hemoglobin level above the normal population values has been considered a hallmark of lifelong adaptation to high-altitude hypobaric hypoxia. Though varied among different geographical locations and populations, an increase in the level of hemoglobin is an acute response (acclimatization) to high-altitude hypoxia for new individuals who have recently moved from sea level [15]. An increase in the concentration of hemoglobin improves blood oxygen-carrying capacity and offsets the hypobaric condition [16].

The landmark research done by Beall et al. on the presence of possible hematological adaptations in Ethiopian high-altitude natives revealed the presence of unique hematological adaptations observed in the Simien Mountain areas of Amhara residents who inhabited for millennia. This unique adaptation marks the presence of the third successful pattern of body adaptations to hypoxia [15]. Amharas did not have elevated hemoglobin (no erythrocytosis) values as compared to their seal level counterparts. There was normal hemoglobin saturation and arterial oxygen level despite hypobaric conditions. Tibetans, unlike Amhara populations, had arterial hypoxemia but not erythrocytosis [17], whereas Andeans had both erythrocytosis and arterial hypoxemia [18].

Cheong et al. revealed the presence of an alternative adaptive mechanism for chronic hypoxia in these populations. Amhara highlanders did not show elevation of hemoglobin but had elevated NO and cGMP in their blood [19], indicating the possibility of adaptation via vasodilatation enhancing oxygen delivery to metabolic tissues. In their study, surprisingly, contrary to Amharas, the Oromos of Ethiopians native to the Bale Mountains showed an elevation in hemoglobin concentration, highlighting the presence of different adaptive mechanisms for similar hypobaric hypoxia challenges. It is also worth noting that erythropoiesis genes were strongly expressed in Ethiopians at high altitude and remained significantly higher at sea level [20].

Despite the presence of an unavoidable low ambient partial pressure of oxygen level, a maladaptation to chronic high-altitude hypoxia, chronic mountain sickness (CMS), is common in the Andes, occasionally found in Tibetans, but absent in Ethiopians [20, 21].

It is worth noting, though, that CMS symptoms can exist without an elevated hemoglobin level and that an elevated hemoglobin level can exist without CMS symptoms [22].

### 3.2. Vascular Adaptations

A common adaptive response to hypobaric hypoxia is an increase in the concentration of hemoglobin, though the degree of elevation is minor in the Amharas of the Simien Mountains of Ethiopia as compared to other native highlanders, suggesting the presence of alternative means of adaptation. Investigators found that vascular response is another adaptive mechanism involved in Amhara highlanders to compensate for their weak hemoglobin response [19].

Vasodilatation is an important physiological adjustment to increase the tissue blood flow in response to hypoxia [23]. Tibetans, like Amharas, have a dampened hemoglobin response to hypoxia and have been recognized to have high nitric oxide levels and enhanced tissue blood flow [17, 24]. NO is a key molecule involved in vasodilation and the prevention of thrombosis. Hypoxic-induced systemic vasodilation is attributed to the enhanced production of NO and has been noticed in the Tibetans. Native Amhara highlanders were also shown to have more NO and its downstream signal transducer cGMP, which was not the case for their Oromo counterparts [19].

In the Amhara highlanders, chronic hypoxia induced elevated NO, enabling vasodilation and an increase in blood flow, compensating for their relatively lower hemoglobin response [19]. In addition, to ensure better and maintained tissue blood flow, vascular endothelial growth factor C (VEGFC), which is crucial for angiogenesis in response to hypoxia, was very high in Ethiopians both at high altitude and in the lowlands [20].

At high altitudes, the entire lung is unavoidably exposed to lowered inspired oxygen. An immediate reaction is the hypoxic pulmonary vasoconstriction reflex, measured as an increase in pulmonary arterial blood pressure that automatically increases pulmonary vascular resistance in poorly aerated regions of the lungs, thereby redirecting pulmonary blood flow to regions richer in oxygen content [25]. This diversion of blood flow to better ventilated alveoli is critical for matching ventilation with perfusion, decreasing the volume of shunted blood and thereby preventing hypoxemia. Chronic hypoxia promotes pulmonary vasoconstriction and increases pulmonary arterial pressure and arterial resistance, which ultimately leads to right ventricular hypertrophy and eventually heart failure.

A subset of chronic mountain sickness, chronic high-altitude pulmonary hypertension (HAPH), is a clinical syndrome seen in individuals residing in high-altitude regions and is characterized by increased pulmonary vascular resistance secondary to hypoxia-induced pulmonary vasoconstriction and vascular remodeling of pulmonary arterioles [26, 27]. The vascular alterations involve all elements of the vessel wall and include endothelial dysfunction, smooth muscle extension into previously nonmuscular vessels, and adventitial thickening [27].

The prevalence of HAPH is influenced by the altitude, ethnicity, and ancestral history of colonization to high altitudes and the presence of underlying cardiorespiratory diseases. The most important risk factors for HAPH were increasing age, hypoxemia, and erythrocythemia [28]. Tibetans are considered to be the most adapted to the stress of high altitude and have the least prevalent HAPH compared to Andeans [29]. Chronic hypoxia promotes angiogenesis by modulating the transcriptional regulator hypoxia-inducible factor 1 alpha (HIF-1*α*), which in turn triggers the upregulation of the erythropoietin [30]. HIF-1*α* is a master regulator of the hypoxic response, and its proangiogenic activities include regulation of vascular endothelial growth factor (VEGF), but also erythropoietin and its receptors (EpoR) [31, 32].

Chronic hypoxia caused by the migration of native sea-level dwellers to high altitudes or chronic lung disease leads to the development of increased pulmonary vascular resistance and pulmonary hypertension [33]. The classical model to show the presence of pulmonary hypertension is increased vascular resistance. In contrast to this general consensus, the Ethiopian study found that Amhara natives at 3700 m had elevated pulmonary artery pressure (27.9 ± 8.4 mmHg), but without elevated pulmonary vascular resistance. It has been suggested that the high pulmonary artery pressure could be due to high pulmonary blood flow and right ventricular enlargement but not to vascular resistance [34]. This finding suggests the pulmonary vasculature may respond to hypoxia in a distinct way.

Another yet unique feature of Amhara highlanders is the sensitivity of their brains to oxygen shortages. The brain is the most sensitive organ to a shortage of oxygen. Many symptoms of chronic mountain sickness are possibly linked with this neuronal hypoxia. One sign of the failure to adapt to acute or chronic hypobaric hypoxia is the presence of severe high-altitude cerebral edema (HACE) [35, 36]. Physiologically, CO_2_ has a cerebrovascular dilation effect, whereas oxygen has the opposite. Cerebral vasculatures exposed to chronic hypoxia are thus at risk of constriction, resulting in decreased blood flow.

The cerebral circulation of Amhara highlanders was found to be less sensitive to hypoxia, unlike in Peruvian counterparts [20]. Amhara highlanders present with high sensitivity of cerebral blood vessels for CO_2_ which would likely increase cerebral blood flow and increase oxygen delivery to the brain. This unique feature makes them better suited for survival at high altitudes.

## 4. Limitation of the Review

This review is not without limitations. First, I did not include the findings regarding the genetic architecture of Ethiopians and the influence of genetic variance on the adaptation mechanisms. Second, I did not comprehensively present each and every similarity and difference between Ethiopian highland natives' and others' coping mechanisms for high-altitude hypoxia, for which the reader might not be satisfied. I encourage readers to approach the excellent references cited in this paper.

## 5. Conclusion

Amharas native to the Simien Mountains of Ethiopia have no signs of chronic mountain sickness, an indicator of a failure of adaptation to chronic high-altitude hypoxia. They have no elevated hemoglobin (contrary to Oromos), indicating that there is no erythrocytosis for hypoxia response and, despite the low oxygen level in the atmosphere, there is no arterial hypoxemia.

Native Amhara highlanders did show elevated NO and cGMP, indicating the possibility of adaptation via vasodilatation enhancing oxygen delivery to metabolic tissues. It was suggested that these unique adaptation mechanisms may be linked to genetic background. Further research is warranted to translate these background natural features of Ethiopian native highlanders to clinical applications.
